# Gene regulatory network resource aids in predicting trans-acting regulators of biosynthetic gene clusters in *Aspergillus fumigatus*

**DOI:** 10.1128/mbio.03874-24

**Published:** 2025-02-18

**Authors:** Hye-won Seo, Jin Woo Bok, Nancy P. Keller

**Affiliations:** 1 Department of Medical Microbiology and Immunology, University of Wisconsin—Madison, Madison, Wisconsin, USA; 2 Department of Plant Pathology, University of Wisconsin—Madison, Madison, Wisconsin, USA; Yonsei University, Seoul, South Korea

**Keywords:** *Aspergillus fumigatus*, secondary metabolism, global regulatory networks, biosynthetic gene cluster, trans-acting regulator

## Abstract

The field of secondary metabolism has greatly benefitted from computational advances in recent years. This has been particularly true for fungal natural product studies. Strides in genome mining have led to the identification of an extraordinary number of secondary metabolite biosynthetic gene clusters (BGCs) across the fungal Kingdom and metabologenomic platforms can group BGCs into gene cluster families and link them to initial chemical structures. Missing are computational applications focused on identifying BGC regulatory networks. Here, we applied the new online gene regulatory network resource, GRAsp (Gene Regulation of *Aspergillus fumigatus*), to identify unknown and unpredictable BGC trans-acting transcriptional/metabolite production modules. GRAsp correctly predicted a two-component regulatory module composed of the transcription factors (TFs), RogA (regulation of gliotoxin) and HsfA, which negatively regulate the gliotoxin BGC and are also involved in gliotoxin self-protection. RogA functions through the repression of *gliZ,* the pathway-specific gliotoxin TF, and HsfA functions by activating *rogA* expression. Furthermore, GRAsp identified TFs that regulate the production of two BGCs lacking pathway-specific TFs, the helvolic acid and fumitremorgin BGCs, respectively. Finally, the known TF, NsdD, was predicted and found to regulate the hexadehydroastechrome BGC. These advances highlight the power of inference algorithms to uncover unpredictable networks in specialized metabolite synthesis.

**IMPORTANCE:**

Toxic secondary metabolites are virulence factors of the opportunistic fungal pathogen *Aspergillus fumigatus,* yet the transcriptional networks regulating secondary metabolite production remain elusive. Uncovering novel regulators without any prior information is challenging. Computational programs have gained prominence in the field of secondary metabolite research due to their accuracy and ability to handle vast amounts of data, including DNA, RNA, and protein data. In this study, a newly developed online computer platform, Gene Regulation of *A. fumigatus*, was used to identify five regulators involved in the production of several *A. fumigatus* toxins, including gliotoxin, helvolic acid, fumitremorgin, and hexadehydroastechrome. This work illustrates the potential for discovering new trans-acting regulators and mechanisms of secondary metabolite regulation through the examination of computational gene regulatory networks.

## INTRODUCTION

Fungi, including *Aspergillus fumigatus*, produce numerous secondary metabolites (SMs), providing fitness attributes such as pigmentation to protect spores from UV damage, acquisition of essential micronutrients, and defensive compounds during interactions with other organisms (1, 2). Fungal SMs have also been invaluable as platforms for developing front-line drugs, including innovative therapeutic agents for infectious diseases, lipid disorders, cancer, and immunomodulation (3, 4). In general, the genes involved in biosynthesis, modification, and transport of any single SM are arranged sequentially in discrete loci termed biosynthetic gene clusters (BGCs). Most fungal BGCs contain at least one core gene encoding a chemical class-defining enzyme that synthesizes the scaffold of the secondary metabolite. The core enzyme usually belongs to one of four major classes: non-ribosomal peptide synthetases (NRPS), polyketide synthases (PKS), terpene cyclases/synthases, and isocyanide synthases (5, 6). There are also hybrid variants of fungal SMs obtained by combining different classes of scaffolds, including NRPS/PKS hybrids, NRPS/terpenoids, PKS/terpenoids, and alkaloid/terpenoids (7). In addition to the core enzymes, the BGC includes additional genes encoding tailoring enzymes that modify the synthase backbone (e.g., oxidases, hydroxylases, methyltransferases, P450 monooxygenases, epimerases, etc.) and transporters involved in intermediate and/or final product transport (2). In certain BGCs, a gene encodes a protein that provides resistance or protection from endogenous toxic SMs (8).

A key gene in terms of SM discovery is the BGC pathway-specific transcription factor (PSTF). Genetic overexpression (OE) of such transcription factors (TFs) generally results in high SM production (9–11). However, not all BGCs contain PSTFs. A recent review of known *Aspergillus* BGCs showed the presence of PSTFs to be strongly correlated with the size of the BGC, typically found with those BGC containing more than five genes (12). The mechanisms regulating smaller BGCs are largely unknown, although some have been linked to partial regulation by “Global TFs” that respond to environmental stimuli. For example, the *A. fumigatus* spore SM, endocrocin, is encoded by a four-gene BGC. Transcriptional activation of the endocrocin BGC requires cascade activation by two global TFs where LaeA (known as a global regulator of SMs) activity removes heterochromatic marks on the promoter of *brlA* encoding the TF required for conidiophore formation (13). Only when BrlA is active, the endocrocin BGC significantly expressed with concomitant metabolite production. These complex cascades are also important for the expression of larger BGCs, even those containing PSTFs. This is most clearly seen in studies of AreA (global regulator of nitrogen metabolism) and HapX/SreA (global regulators of iron homeostasis), both of which regulate many BGCs, including those with PSTFs (14, 15).

Unlike the near certainty of the role of PSTFs in BGC regulation, the impact of global regulators and environmental factors on BGC expression and subsequent SM production is unpredictable. To address this bottleneck, we sought to examine the potential of computational innovation to advance an understanding of the foundations of regulation logic of fungal SM. Thus, we recently developed a computational inference algorithm to establish a genome-wide gene regulatory network using *A. fumigatus* as our first model. This work generated a user-friendly online resource named Gene Regulation of *Aspergillus fumigatus* (GRAsp). Our first assessment of GRAsp uncovered a trans-acting TF, regulator of gliotoxin (RogA), negatively regulating the gliotoxin BGC (16). Here, we find GRAsp predictions of trans-acting BGC regulators that are extensive and accurate. We demonstrate the rigor of this program by identifying a second trans-acting regulator of the gliotoxin BGC that connects RogA biosynthetic genes, a trans-acting regulator of the helvolic acid BGC (lacking a PSTF), a trans-acting regulator of the fumitremorgin BGC (also lacking a PSTF) and a trans-acting regulator of the hexadehydroastechrome BGC. These advances highlight the power of inference algorithms to uncover unpredictable production networks in specialized metabolite synthesis.

## RESULTS

### GRAsp identifies trans-acting regulators of the gliotoxin BGC

**Fig 1:**
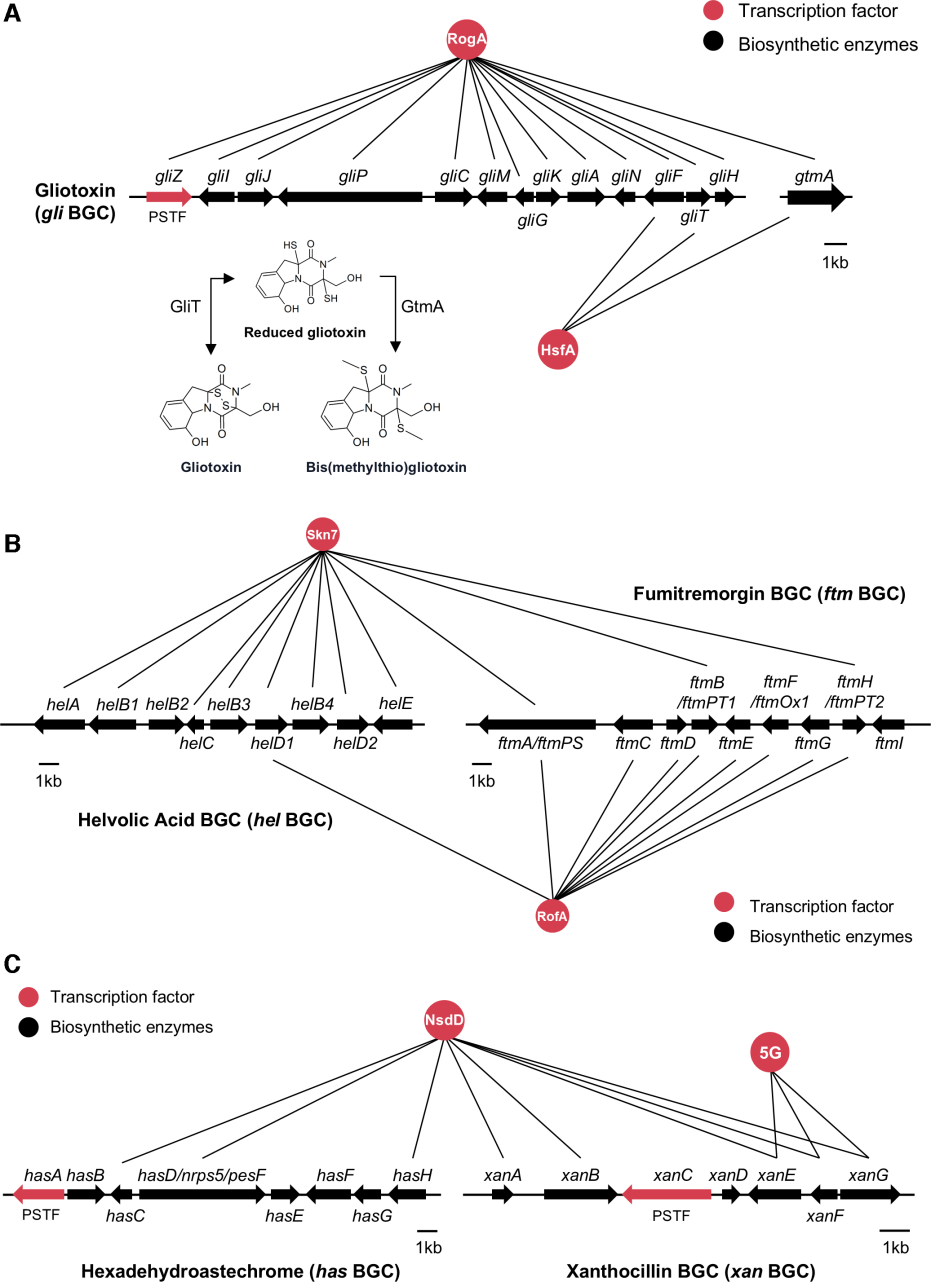
Three GRAsp models of predictive linkage of regulators and SM biosynthetic clusters. (**A**) RogA and HsfA are predicted to regulate gliotoxin synthesis. GliT and GtmA are self-protection enzymes modifying gliotoxin structure as shown. (**B**) Skn7 and RofA are predicted to regulate helvolic acid and fumitremorgin synthesis. (C) NsdD is predicted to regulate hexadehydroastechrome and xanthocillin synthesis. AFUA_5G02800 (5G) is predicted to regulate xanthocillin synthesis. Lines connect TFs to genes they are predicted to regulate, as predicted by GRAsp.

GRAsp is an online resource that allows for the visualization and exploration of a gene regulatory network previously inferred from gene expression data sets. One prominent feature of GRAsp is that it groups co-expressed and co-regulated genes into clusters called modules. Previously, this resource, along with its module feature, was used to identify a previously uncharacterized TF (AFUA_3G11990), which we termed RogA, and found to be a negative regulator of gliotoxin (16). However, we did not assess if RogA regulated the expression of *gliT* and *gtmA*, encoding proteins with direct and indirect self-protection against gliotoxin, respectively (17, 18), or if a second TF in the GRAsp prediction, HsfA (heat shock factor, AFUA_5G01900), was also involved in their regulation as suggested by GRAsp (Fig. 1A) (16).

**Fig 2:**
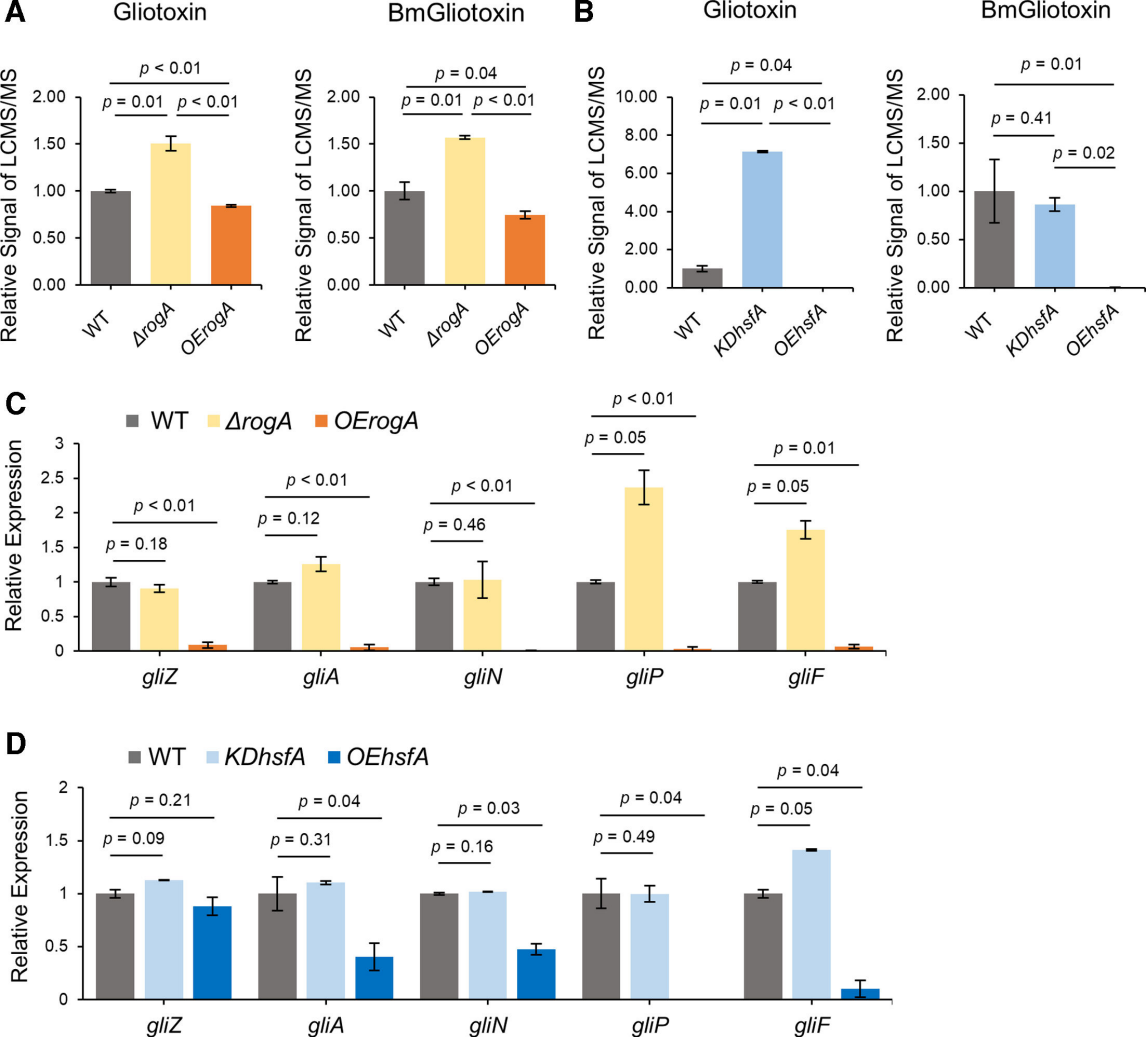
OE of RogA and HsfA inhibits gliotoxin biosynthesis. (**A and B**) Gliotoxin and bis(methylthiol)gliotoxin (BmGliotoxin) were quantified in deletion (**D**), OE, or KD mutants and compared to production in the wild type (set at 1). (**C and D**) The semi-quantitative reverse transcription and PCR analysis of a subset of gliotoxin biosynthetic genes and the gliotoxin PSTF *gliZ* was compared between mutants and wild type (set to 1). All *P*-values were reported to two decimal places, and values less than 0.01 were expressed as *P* < 0.01.

HsfA was first characterized in the *A. fumigatus* A1160 background, where it was found to be an essential protein required for thermotolerance (19). To stay in the same background as the *rogA* mutants, we created both a knockdown (KD) strain of *hsfA* (KD*hsfA* as deletion of *hsfA* was lethal [19]) and an overexpression strain (OE*hsfA*) in *A. fumigatus* Af293 (Fig. S1). Next, we compared the production of gliotoxin and its predominant derivative, bis(methylthio)gliotoxin, which is produced by GtmA, in the two *hsfA* mutants to WT, Δ*rogA*, and OE*rogA*. As found before (16), comparative analysis via ultra-high-performance liquid chromatography-high resolution mass spectrometry (UHPLC-HRMS/MS) revealed an increase in gliotoxin/bis(methylthio)gliotoxin levels in Δ*rogA* and a decrease in gliotoxin/bis(methylthio)gliotoxin levels in OE*rogA* compared to WT (Fig. 2A; Table S1). We also found that gliotoxin production was increased in the KD*hsfA* strain, although bis(methylthio)gliotoxin production was not significantly decreased in KD*hsfA* compared to WT (Fig. 2B; Table S1). The *OEhsfA* strain did not produce either metabolite.

To determine whether RogA and HsfA transcriptionally regulate gliotoxin synthesis, the RNA levels of a subset of the gliotoxin biosynthetic genes (*gliZ*, *gliA*, *gliN*, *gliP*, and *gliF*) were measured. Both OE*rogA* and OE*hsfA* inhibited the expression of the enzymatic *gli* genes (Fig. 2C and D). Interestingly, only OE*rogA* inhibited the expression of *gliZ* encoding the gliotoxin PSTF (Fig. 2C). Taken together, the transcript and chemical data show that both RogA and HsfA function as negative regulators in gliotoxin synthesis.

### RogA-dependent gliotoxin regulation occurs through the regulation of the gliotoxin pathway-specific transcription factor GliZ

**Fig 3:**
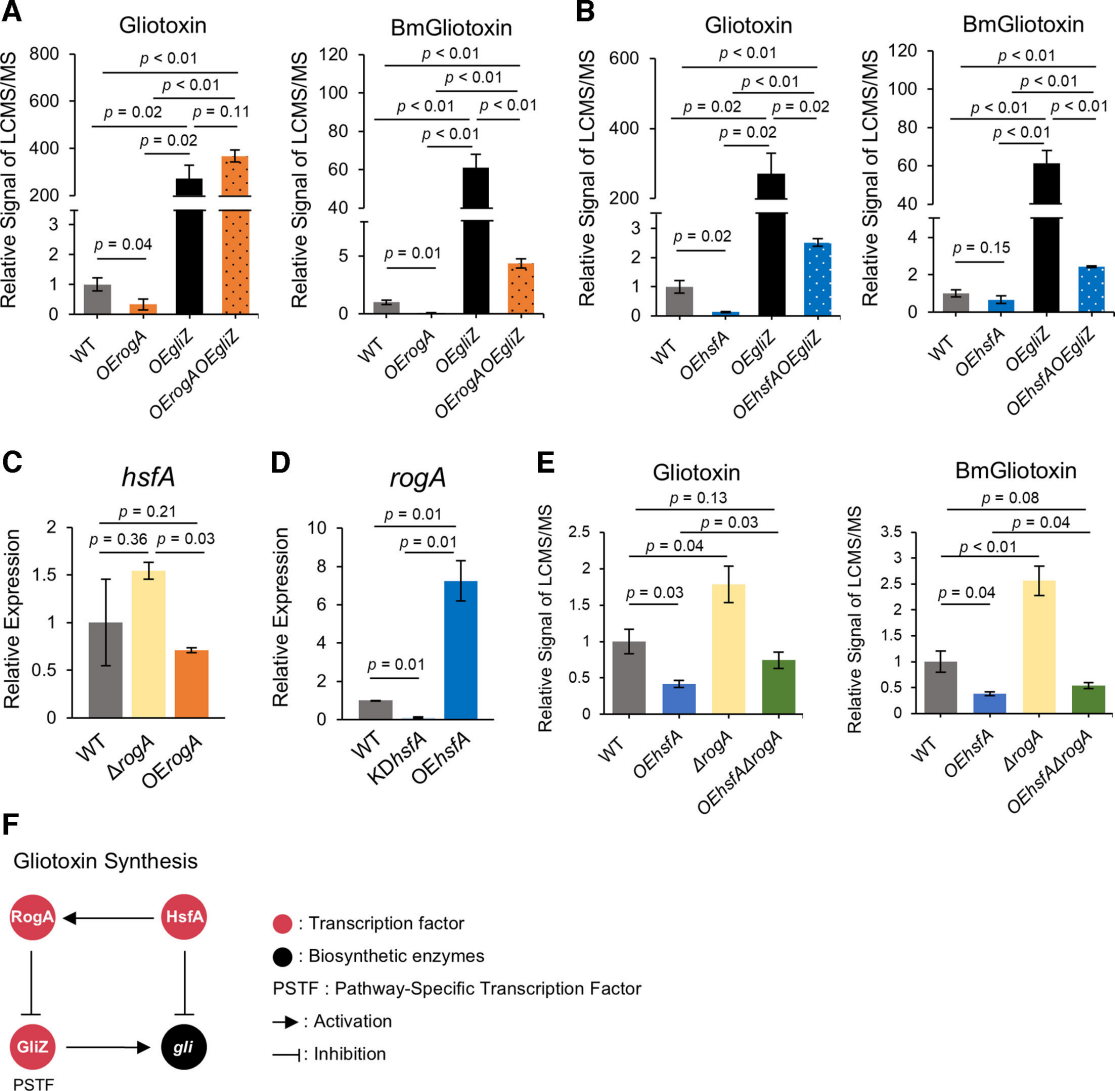
An interconnection of GliZ, RogA, and HsfA in regulating gliotoxin. (**A and B**) OE of *gliZ* restores gliotoxin and BmGliotoxin in OE*rogA* and OE*hsfA*. Gliotoxin and BmGliotoxin were quantified in OE and double OE mutants and compared to production in the wild type (set at 1). (**C–E**) Gliotoxin suppression in OE*hsfA* is partially through HsfA-positive regulation of RogA. The semi-quantitative reverse transcription and PCR analysis of *hsfA* (**C**) and *rogA* (**D**) was compared between mutants and wild type (set to 1). Gliotoxin and BmGliotoxin were quantified in OE and double OE, D mutants, and compared to production in the wild type (set at 1). All *P*-values were reported to two decimal places, and values less than 0.01 were expressed as *P* < 0.01. (**F**) A model summarizing the interconnection of GliZ, RogA, and HsfA in gliotoxin regulation.

To address the finding that RogA but not HsfA negatively regulated *gliZ*, we hypothesized that overexpressing *gliZ* could rescue gliotoxin synthesis in OE*rogA* but not OE*hsfA*. Three strains were created to address this hypothesis: OE*gliZ* and two double mutants OE*rogA*OE*gliZ* and OE*hsfA*OE*gliZ* (Fig. S2). A comparison of gliotoxin/bis(methylthio)gliotoxin levels showed that gliotoxin production was similar in the *OEgliZ* and the OE*rogA*OE*gliZ* strain, more than 100-fold over WT (Fig. 3A). Although there was an increase in gliotoxin synthesis in the OE*hsfA*OE*gliZ* strain compared to both WT and OE*hsfA*, this increase was only ca. twofold (Fig. 3B), suggesting that while RogA inhibits gliotoxin production primarily through *gliZ*, HsfA has an alternative pathway for repressing gliotoxin production independent of GliZ.

### HsfA is a positive regulator of RogA

Since both RogA and HsfA were placed in the same GRAsp module (Fig. 1A), we thought it is possible that they could regulate each other’s expression. Neither deletion nor overexpression of *rogA* statistically changed *hsfA* expression compared to WT, although there was decreased *hsfA* expression in comparing *OErogA* to Δ*rogA* (Fig. 3C). In contrast, *rogA* expression was significantly downregulated in KD*hsfA* and upregulated in OE*hsfA* compared to WT (Fig. 3D). To examine the extent of RogA involvement in HsfA regulation of gliotoxin synthesis, an OE*hsfA*Δ*rogA* double mutant was created (Fig. S3), and gliotoxin/bis(methylthio)gliotoxin production level was compared with that of WT, OE*hsfA*, and Δ*rogA*. Both gliotoxin and bis(methylthio)gliotoxin production in OE*hsfA*Δ*rogA* was higher than in OE*hsfA*, and the metabolite abundance restored to nearly WT levels in the double mutant (Fig. 3E). In summary, gliotoxin synthesis is regulated by a pathway comprising HsfA, RogA, and GliZ, as well as an alternative pathway where HsfA appears to act independently (Fig. 3F).

### Transient impact of RogA but prolonged impact of HsfA on exogenous gliotoxin resistance

**Fig 4:**
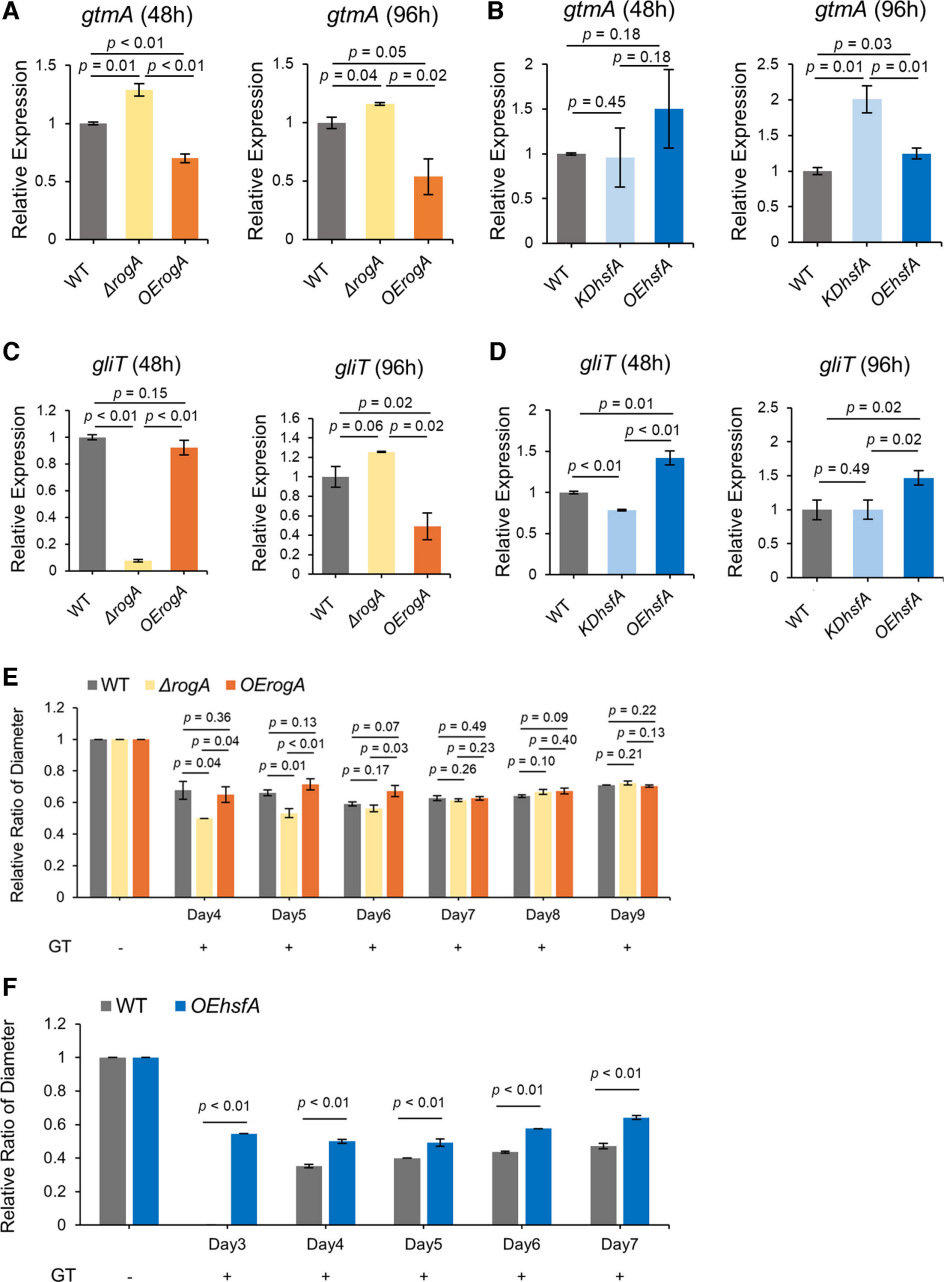
RogA and HsfA regulation of *gliT* correlates with resistance to exogenous gliotoxin. (**A–D**) The semi-quantitative reverse transcription and PCR analysis of *gtmA* and *gliT* was compared between RogA and HsfA mutants and wild type (set to 1). (**E and F**) Diameter of *rogA* mutants (**E**) and OE*hsfA* mutant (**F**) was compared to wild type (set to 1 in no gliotoxin treatment) over successive days. Gliotoxin was added at 50 µg/mL. All *P*-values were reported to two decimal places, and values less than 0.01 were expressed as *P* < 0.01.

As shown in Fig. 1A, GRAsp also predicted that both RogA and HsfA regulate *gliT* and *gtmA*. GliT is considered a direct self-protection protein by neutralizing the toxicity of gliotoxin, whereas GtmA indirectly provides self-protection by converting reduced gliotoxin to the less toxic bis(methylthio) gliotoxin (18). RNA expression results indicated that RogA negatively regulated *gtmA* expression (Fig. 4A) but had a differential impact on *gliT* over time, where it appeared important for early *gliT* expression (Fig. 4C). HsfA did not regulate *gtmA* in a consistent way (Fig. 4B) but appeared to be a positive regulator of *gliT* (Fig. 4D).

Because GliT is an essential enzyme required for self-defense against exogenous gliotoxin and both HsfA- and RogA-regulated *gliT* expression, we hypothesized that HsfA and/or RogA could also be involved in self-defense against exogenous gliotoxin. To determine if RogA impacted *A. fumigatus* resistance to exogenous gliotoxin, WT, Δ*rogA*, and OE*rogA* were point-inoculated on glucose minimal media (GMM) with or without gliotoxin, and their diameters were measured daily. Figure 4E (and Fig. S4) shows the relative ratios of the diameters of strains treated with gliotoxin compared to the gliotoxin-untreated control. All strains began to grow 4 days after inoculation in the gliotoxin treatment. The Δ*rogA* showed significantly slower growth than both WT and OE*rogA* strains on days 4 and 5, but by day 6, Δ*rogA* grew similarly to WT, and all strains were comparable on days 7–9. Based on this data, it appears that RogA may provide initial but not long-term protection against exogenous gliotoxin, which matches with *gliT* gene expression in the Δ*rogA* strain (Fig. 4C).

A similar procedure was used to ask if HsfA overexpression affected resistance to exogenous gliotoxin (the knockdown strain grew so poorly in glucose media such that it was not possible to get reliable data). Growth of the *OEhsfA* strain was observed 3 days after inoculation, while WT started to grow 4 days after inoculation. Additionally, the diameters of *OEhsfA* were greater than those of WT throughout the experiment period (Fig. 4F; Fig. S5). This indicates that OE*hsfA* exhibited higher resistance to exogenous gliotoxin compared to WT, which correlates to HsfA regulation of *gliT* (Fig. 4D).

### Additional secondary metabolite modules predicted by GRAsp

Several other trans-acting TFs were predicted to regulate other *A. fumigatus* BGCs from GRAsp (Fig. 1). In particular, Skn7 (AFUA_6G12522) and AFUA_1G14945 were both linked to the helvolic acid and fumitremorgin BGCs, sharing proposed regulation of one *hel* gene (*helD1*) and three *ftm* genes (*ftmA, ftmB* and *ftmH*) (Fig. 1B). NsdD (AFUA_3G13870) was linked with the hexadehydroastechrome and xanthocillin BGCs (Fig. 1C). Helvolic acid and fumitremorgin BGCs are somewhat unusual for their size (both a nine-gene BGC [20]) in that neither BGC contains a PSTF. Typically, BGCs with five or more genes also contain a PSTF (12). Helvolic acid is a terpene-derived mycotoxin, and fumitremorgin is a prenylated indole alkaloid mycotoxin. The hexadehydroastechrome and xanthocillin BGCs both contain PSTFs (21, 22).

**Fig 5:**
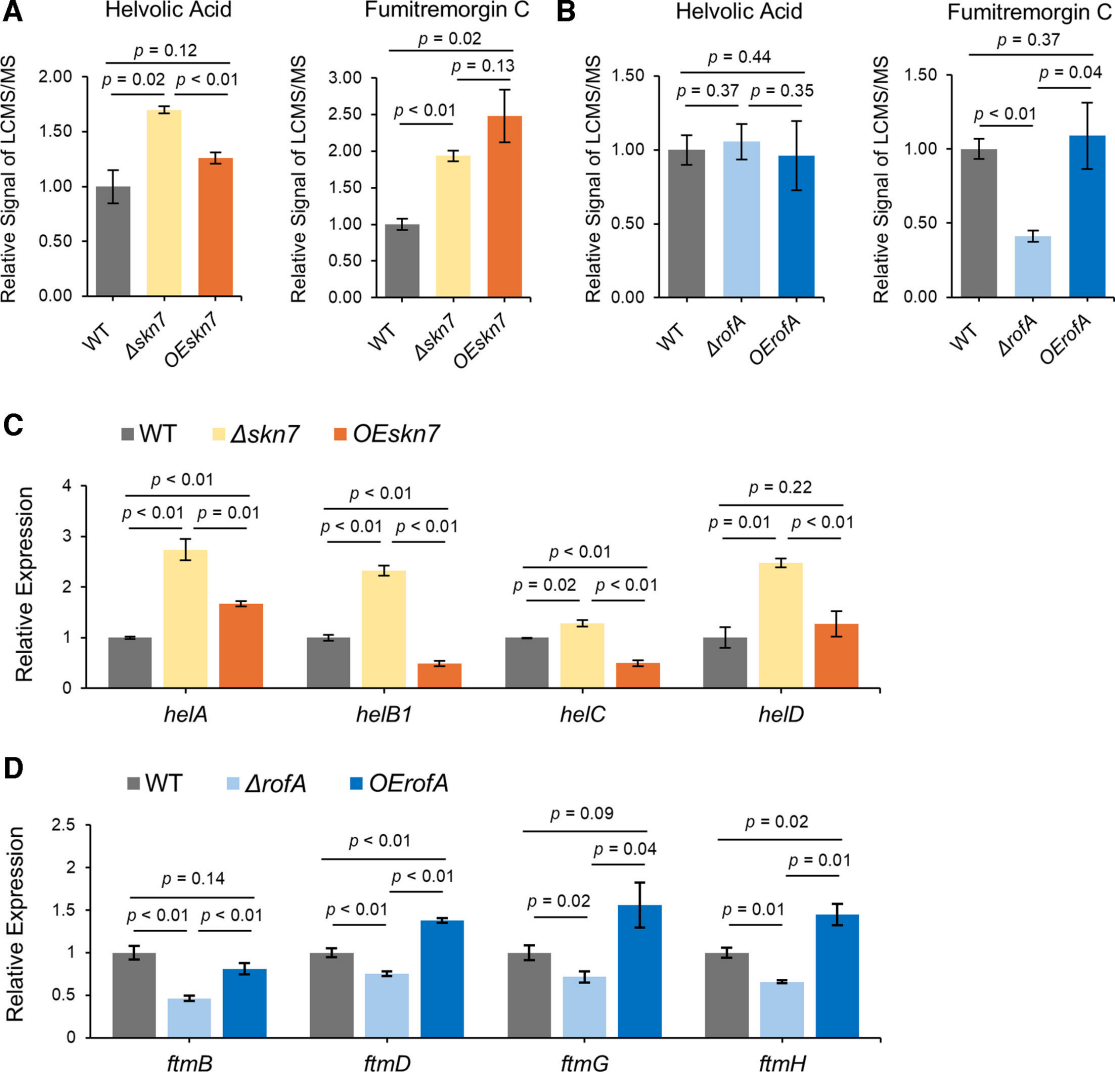
Skn7 and RofA are involved in synthesis of helvolic acid and fumitremorgin C. (**A**) Helvolic acid and fumitremorgin C were quantified in deletion (**D**) and OE *skn7* mutants and compared to production in the wild type (set at 1). (**B**) Helvolic acid and fumitremorgin C were quantified in deletion (**D**) and OE *rofA* mutants and compared to production in the wild type (set at 1). All *P*-values were reported to two decimal places, and values less than 0.01 were expressed as *P* < 0.01. (**C and D**) The semi-quantitative reverse transcription and PCR analysis targeting four genes of helvolic acid BGC and fumitremorgin BGC, respectively, was performed comparing mutants and wild type (set to 1). All *P*-values were reported to two decimal places, and values less than 0.01 were marked as *P* < 0.01.

To determine whether Skn7 is involved in the synthesis of helvolic acid or/and fumitremorgin, we created Δ*skn7* and OE*skn7* mutants (Fig. S6), which were grown along with WT for 5 days on liquid GMM. Culture extracts were analyzed using UHPLC-HRMS/MS. The chemical analysis showed that the amount of helvolic acid was significantly increased in Δ*skn7* compared to WT, whereas the OE*skn7* strain showed no difference to WT. Both strains showed an increase in fumitremorgin synthesis compared to WT (Fig. 5A; Table S2). Transcriptional analysis of the nine genes of *hel* BGC, comparing transcript levels in *skn7* mutants to WT, showed that expression of four genes (*helA*, *helB1*, *helC*, and *helD1*) increased in Δ*skn7*, while their levels either returned to that of the WT or decreased further in OE*skn7* (Fig. 5C; Table S3). These results are consistent with Fig. 5A, suggesting that Skn7 imparts some negative control over helvolic acid production. Expression of *skn7* did not affect the transcript levels of the *ftm* BGC (Table S2), aligning with fumitremorgin data in Fig. 5A. The deletion and overexpression of *AFUA_1* G14945, which we term RofA (regulator of fumitremorgin; Fig. S7), gave a clearer result. WT, Δ*rofA*, and OE*rofA* strains were grown as for the Skn7 mutants, and their chemical profiles were examined. Neither deletion nor overexpression of *rofA* affected helvolic acid production. However, fumitremorgin C synthesis was significantly reduced in *ΔrofA* compared to WT (Fig. 5B; Table S2). There was no effect of OE*rofA* on fumitremorgin C production. Among the nine genes of the *ftm* BGC, four genes (*ftmB*, *ftmD*, *ftmG*, and *ftmH*) were downregulated in Δ*rofA* but upregulated in OE*rofA* (Fig. 5D; Table S3), which aligns with the chemical results. These results suggest that RofA is required for the full production of fumitremorgin.

**Fig 6:**
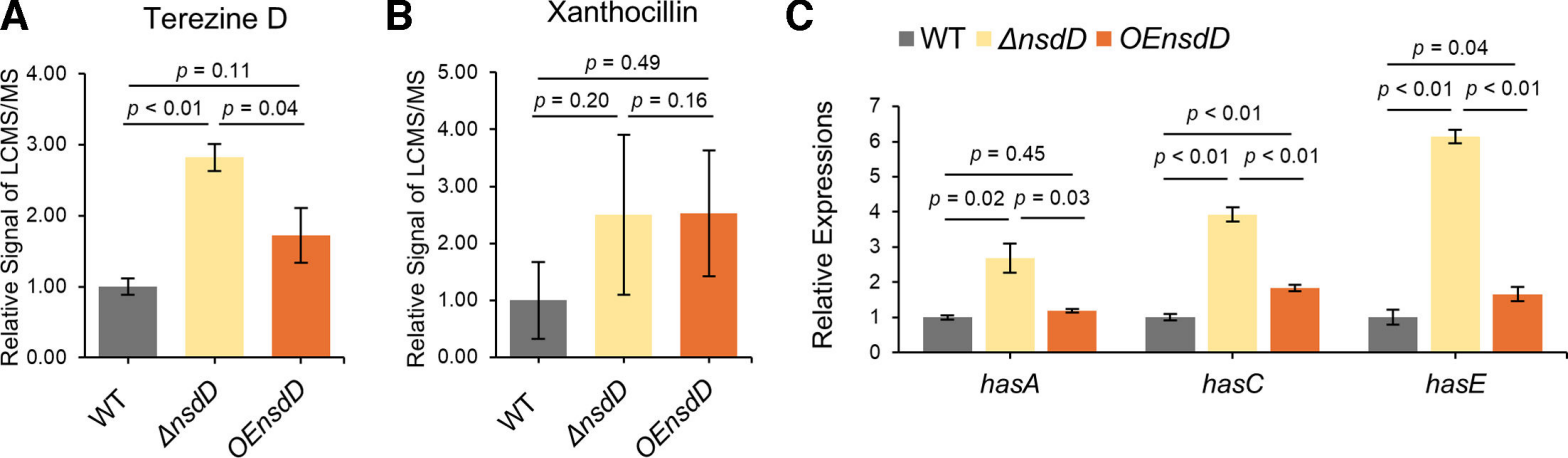
NsdD negatively regulates terezine D synthesis but does not affect the synthesis of xanthocillin. (**A and B**) Terezine D (**A**) and xanthocillin (**B**) were quantified in deletion (**D**) and OE *nsdD* mutants and compared to production in the wild type (set at 1). All *P*-values were reported to two decimal places, and values less than 0.01 were expressed as *P* < 0.01. (**C**) The semi-quantitative reverse transcription and PCR analysis was performed to detect genes of hexadehydroacetochrome BGC in mutants and wild type (set to 1). All *P*-values were reported to two decimal places, and values less than 0.01 were marked as *P* < 0.01.

Surprisingly, while the UHPLC-LCMS/MS data showed no impact of RofA on helvolic acid production, eight out of nine genes in the *hel* BGC were negatively affected by *rofA* expression (Table S3), suggesting a strong relationship between *rofA* and helvolic acid. The intensity of the peaks obtained through UHPLC-LCMS/MS was sufficient for clear measurement (Table S3). Therefore, we consider that there are no concerns regarding the extraction method for helvolic acid or potential analytical errors from the UHPLC-LCMS/MS equipment. It is possible that the timing of helvolic acid extraction in this experiment was not appropriate for assessing the impact of RofA on helvolic acid. The final module we examined addressed the GRAsp prediction that NsdD, already characterized for its involvement in both asexual and sexual sporulation processes (23–25), could be involved in hexadehydroastechrome and xanthocillin biosynthesis. As shown in Fig. 1C, NsdD is connected to three hexadehydroastechrome BGC genes (*hasC*, *hasD*, and *hasH*) and five xanthocillin BGC genes (*xanA*, *xanB*, *xanE*, *xanF*, and *xanG*) but, interestingly, lacks a connection to both the hexadehydroastechrome and the xanthocillin BGC PSTFs, *hasA* and *xanC*, respectively (21, 26). After creating the Δ*nsdD* and OE*nsdD* mutants (Fig. S8), we compared target metabolites in extracts of WT, Δ*nsdD*, and OE*nsdD* strains cultured for 5 days in liquid GMM. Since hexadehydroastechrome is difficult to quantify, we instead measured its stable precursor, terezine D. As depicted in Fig. 6A and Table S4, terezine D production was significantly increased in Δ*nsdD* compared to WT. The OE*nsdD* strain produced WT levels of terezine D. We next chose five genes in the *has* BGC (*hasA*, *hasC*, *hasD*, *hasE*, and *hasG*) for transcriptional analysis and found that *hasA*, *hasC*, and *hasE* were upregulated in Δ*nsdD* (Fig. 6C; Table S5), matching with the chemical data. However, the GRAsp prediction that xanthocillin production could be dependent on *nsdD* did not bare out, either by chemical analysis (Fig. 6B; Table S4) or transcript analysis (Table S5) in the conditions tested. These results support the role of NsdD in suppressing hexadehydroastechrome synthesis.

## DISCUSSION

Elucidating regulatory networks in fungal secondary metabolite synthesis remains a challenging hurdle. The field of fungal-specific metabolism has exploded in large part due to computational advances in identifying BGCs and grouping them into gene cluster families (27–29). To mine a fungal genome for drug discovery, the BGC can be heterologously expressed in a model organism, such as *Aspergillus nidulans* (30). To understand how and when endogenous BGCs are activated, target genes in the BGC can be deleted or overexpressed, and subsequent mutants are chemically analyzed for lack or increased production of the BGC product (20, 31). For BGCs containing PSTFs, studies often explore how the PSTF is regulated. For instance, how the PSTF AflR is regulated in the aflatoxin/sterigmatocystin BGCs led to the discovery of the global secondary metabolite regulator LaeA (32). However, beyond finding PSTFs and identification of a few global regulators, the identification of trans-acting BGC transcriptional activators has been minimal. Here, we used a computation network program to identify trans-acting TFs of known *A. fumigatus* BGCs, including gliotoxin BGC synthesis.

Gliotoxin is one of the most studied *A. fumigatus* secondary metabolites as it is a virulence determinant (17). The *gli* BGC was thoroughly characterized in a series of studies from 2005 to 2010 (33, 34), including its regulation by the PSTF GliZ in 2006 (35). GliT and GtmA functions were described in 2010 (34, 36) and 2014 (37), respectively. Other proteins known to impact gliotoxin synthesis/resistance include the kinase MpkA (38), the transcription factor RglT (39), and the RGS protein FlbA (40). These proteins act through direct or indirect interactions with either the *gli* BGC or GliT/GtmA. Hence, the roles of GliZ, GliT, and GtmA are key proteins in the production of the compound. The fact that GRAsp predicted that two TFs linked to both *gliT* and *gtmA* genes was particularly motivating for our current study.

Figure 3F summarizes our findings on how RogA and HsfA regulate gliotoxin synthesis. RogA negative regulation of *gliZ* expression leads to a decrease in gliotoxin production, which can be completely remediated by overexpression of *gliZ* in the RogA overexpression strain (Fig. 3A). HsfA-positive regulation of *rogA* expression partially explains HsfA impact on gliotoxin production, although HsfA appears to also negatively regulate gliotoxin synthesis in a RogA/GliZ to be independent manner determined by the minimal impact of OE*gliZ* in the OE*hsfA* background (Fig. 3B). This discovery is significant not only for identifying two new gliotoxin regulators but also for potentially uncovering new pathways for gliotoxin production not mediated by GliZ.

GRAsp also predicted that both RogA and HsfA could regulate the two gliotoxin self-protection genes, *gliT* and *gtmA*. Our gene expression data supported this prediction, particularly for *gliT* (Fig. 4A through D). Because *gliT* deletion mutants are increased in susceptibility to exogenous gliotoxin (34, 41), we hypothesized that HsfA and/or RogA could play a role in gliotoxin resistance. In particular, the OE*hsfA* mutant which showed consistent positive regulation of *gliT* was more resistant to exogenous gliotoxin (Fig. 4E and F). Although GliZ is well recognized as a *gli* BGC PSTF, its regulatory range is not absolute. For example, Schrettl et al. previously reported GliZ-independent expression of *gliT* (21), which may support the role of an HsfA/GliT connection as a previously unreported mediator of self-protection against exogenous gliotoxin.

The GRAsp program also identified two TFs, Skn7 and RofA, linked to helvolic acid and fumitremorgin BGCs. In contrast to the presence of *gliZ* in the *gli* BGC, neither the helvolic acid nor fumitremorgin BGCs contain PSTFs, thus increasing our interest in finding possible TFs regulating these BGCs. We found that Skn7 was involved in the regulation of both metabolites and in a complex fashion for fumitremorgin. Skn7 is one of two response regulators of the high-osmolarity glycerol (HOG) pathway, which is important for adapting to environmental stress, especially oxidative stress (42), and it is possible that its impact on both metabolites is coupled with signaling linked with the HOG cascade. On the other hand, a function for RofA, a canonical C6 TF, has not been reported in the literature. RofA deletion led to a significant reduction in fumitremorgin synthesis coupled with decreased gene expression (Fig. 5D), which aligns with the GRAsp linkage of this TF to almost the entire *ftm* BGC (Fig. 1B). Despite the data supporting Skn7 and RofA regulation of these BGCs, neither TF presented the typical PSTF pattern where PSTF deletion and overexpression normally result in loss and increase of the BGC metabolite (21, 26).

Although the gliotoxin, helvolic acid, and fumitremorgin models predicted by GRAsp largely align well with chemical results, GRAsp prediction of hexadehydroastechrome and xanthocillin regulation by NsdD was only observed for hexadehydroastechrome and not xanthocillin under the growth conditions of this study (Fig. 6). NsdD is a GATA-type transcription factor involved in cleistothecium formation in *Aspergillus* spp. (25, 43). The deletion of *nsdD* increased hexadehydroastechrome synthesis (Fig. 6A), but neither deletion nor OE*nsdD* mutants had an impact on xanthocillin production (Fig. 6B). Possibly, this was due to the lack of connection of NsdD to the xanthocillin PSTF *xanC* (Fig. 1; Table S5), which is absolutely required for xanthocillin synthesis (22) or the low levels of xanthocillin synthesis in the growth conditions used in this study (Table S4). It is also possible that NsdD could regulate xanthocillin under different growth conditions.

Fungal natural products are well known to provide ecological benefits to the producing microbe in response to varied sets of environmental signals. However, the complexity of these signals and the transcriptional relays they orchestrate are not well understood. To address this void in knowledge, we posited that much as computational programs have launched the identification of BGCs, an appropriate computational inference program could similarly identify regulatory networks. By using the GRAsp platform, we have been able to identify five *A. fumigatus* TFs involved in the regulation of gliotoxin, helvolic acid, fumitremorgin, and hexadehydroastechrome synthesis. The program was particularly useful in identifying a previously unknown two-component gliotoxin regulatory unit. Continued implementation of network-based approaches holds significant promise in uncovering the complex wiring of secondary metabolite regulatory networks.

## MATERIALS AND METHODS

### Growth conditions for collecting spores

The spores of *A. fumigatus* Af293 strains were harvested using 0.1% (vol/vol) Tween 80 and grown at 37°C in GMM with appropriate supplements as previously described (44). For collecting spores of the strains with xylose promoter (p*xylP*), glucose was substituted to 20 g/L xylose. All strains were maintained as glycerol stocks at −80°C.

### Construction of mutants

The deletion strains of *A. fumigatus* were created by replacing the target genes with an *Aspergillus parasiticus pyrG* gene. Two 1 kb DNA fragments, which are right upstream and downstream of each open reading frame of the target genes, were amplified by PCR from genomic DNA of *A. fumigatus* TFYL81.5 and were fused to a 2 kb *pyrG* gene from pJW24 (45) using double-joint (DJ) PCR as previously described (46). As *hsfA* is a lethal gene, a knockdown KD strain of *hsfA* was created, which was generated by inserting a xylose-inducible promoter of *xylP* (p*xylP*) with *A. parasiticus pyrG* (KD*hsfA*). As it was previously reported that 467 bp of *hsfA* promoter should be maintained to avoid growth defects and instability of mutants, p*xylP* was inserted in front of the 467 bp promoter region (18). Fungal transformation was performed with the *pyrG*^−^ auxotroph of *A. fumigatus* (TFYL80.1) following previously described methods (32).

The OE strains of *A. fumigatus* were generated by inserting the promoter region of *gpdA* of *A. nidulans* (p*gpdA*) in front of the target genes. Two 1 kb fragments immediately upstream and downstream of the translational start site were amplified by PCR from Af293 genomic DNA. *A. parasiticus pyrG::A. nidulans* p*gpdA* as the selectable marker and overexpression promoter was amplified from the plasmid pJMP9 (47). These three fragments were fused by DJ PCR. Fungal transformation was carried out in the same manner as for generating knockout (KO) strains.

To create the double mutants OE*rogA*OE*gliZ* and OE*hsfA*OE*gliZ*, the *pyrG* marker was removed from OE*rogA* and OE*hsfA* by transforming with a *pyrG* recycle construct. This construct consists of two 1 kb DNA fragments from the upstream and downstream regions of the *pyrG* marker inserted into OE*rogA* and OE*hsfA*. Transformants were grown on GMM containing 5-fluoroorotic acid (5-FOA) and uracil/uridine. After southern blot analysis confirmed that *pyrG* was deleted OE*rogA* and OE*hsfA* (Fig. S2A through C), these *pyrG*^−^ mutants were then transformed with OE*gliZ* DJ PCR product to create double overexpression strains (Fig. S2B and D). All strains and primers are listed in Tables S6 and S7, respectively.

### RNA extraction and sqRT-PCR analysis

Fungal mycelia were grown in 50 mL liquid GMM in 250 mL Erlenmeyer flasks with 10^6^ conidia/mL at 25°C for 4 days in dark. For RNA extraction, mycelia were collected using cloths made of rayon-polyester, washed with distilled water, and lyophilized overnight. Total RNA was extracted using TRIzol (Invitrogen). The 5 µg of total RNA was treated with 1 µL DNase I (2,000 U/mL), and cDNA was synthesized using an iScript cDNA synthesis kit (Bio-Rad) with 1 µg of DNase I-treated RNA.

Semi-quantitative reverse transcription and PCR (sqRT-PCR) was performed using a template of 1 µg cDNA, 1 µL of 10 pmol forward and reverse primers, respectively, and 0.2 µL Taq polymerase (Bulldog-Bio) in a 25 µL reaction mixture. The PCR was carried out under the following conditions: an initial denaturation at 95°C for 5 min, followed by 30 cycles of 95°C for 30 s, 56°C–60°C (depending on the primers) for 30 s, and 72°C for 30 s, with a final hold at 4°C. After electrophoresis of the PCR products in a 2% gel, the band signals were quantified using ImageJ. An amplicon from gDNA was used as a control to check gDNA contamination in the cDNA. The primers used for sqRT-PCR are listed in Table S7.

### Secondary metabolite extraction and analysis

For gliotoxin extraction, 10^6^ conidia/mL of each strain was cultured in 50 mL liquid GMM in 250 mL Erlenmeyer flasks and inoculated at 25°C for 4 days in the dark at 250 rpm. For other SMs extraction, each strain was cultured for 5 days.

For gliotoxin and its derivatives extraction, the supernatant of culture and chloroform was mixed in a 2:1 ratio and incubated overnight. After incubation, the chloroform layers were collected into new glass tubes and completely air dried. To extract other secondary metabolites, the whole cells and supernatant were lyophilized and crushed into fine powders, and 20 mL of chemical solvents was added. Terezine D (a precursor of hexadehydroastechrome) was extracted using 10% methanol in ethyl acetate, fumitremorgin, and xanthocillin using a 1:1 mixture of methanol and ethyl acetate and helvolic acid using methanol. The chemical solvent was incubated for 12 h, transferred to new tubes, and dried overnight in a fume hood. This procedure was repeated three times. The dried extracts were resuspended in methanol at a concentration of 1 mg/mL (for xanthocillin detection, a concentration of 10 mg/mL). Each sample was then filtered using a 0.2 µm syringe filter. Subsequently, all secondary metabolites were analyzed using UHPLC-HRMS/MS. While most SMs were detected in positive mode, xanthocillin and its derivatives were identified in negative mode in UHPLC-HRMS/MS.

### Gliotoxin resistance assay

A total of 5 × 10^3^ conidia of each strain were point inoculated on GMM solid plates, with or without gliotoxin (50 µg/mL). The plates were incubated at 25°C, and the growth diameters of the strains were measured daily for 7–9 days. The Δ*gliT* (34) strain was used as a control to assess gliotoxin activity. Although the growth of Δ*gliT* was inhibited by 10 µg/mL gliotoxin, we observed a little difference in growth compared to the *rogA/hsfA* mutant at this concentration. To examine clearer growth differences from the mutants, we used 50 µg/mL gliotoxin, a higher concentration than reported in other studies.

### Statistical analysis

All experiments, including the analysis of secondary metabolite production and transcript quantification, were performed in triplicate. Differences between the means of experimental groups were analyzed using Welch’s *t*-test, which the Excel program provides. All *P*-values were reported to two decimal places, and values less than 0.01 were expressed as *P* < 0.01.

## SUPPLEMENTAL MATERIAL

Supplemental figures mbio.03874-24-s0001.pdf Fig. S1 to S8.

Supplemental tables mbio.03874-24-s0002.xlsx Tables S1 to S7.

ASM does not own the copyrights to Supplemental Material that may be linked to, or accessed through, an article. The authors have granted ASM a non-exclusive, world-wide license to publish the Supplemental Material files. Please contact the corresponding author directly for reuse.

## References

[1] Macheleidt J, Mattern DJ, Fischer J, Netzker T, Weber J, Schroeckh V, Valiante V, Brakhage AA. 2016. Regulation and role of fungal secondary metabolites. Annu Rev Genet 50:371–392. doi:10.1146/annurev-genet-120215-035203 27732794

[2] Keller NP. 2019. Fungal secondary metabolism: regulation, function and drug discovery. Nat Rev Microbiol 17:167–180. doi:10.1038/s41579-018-0121-1 30531948 PMC6381595

[3] Clardy J, Walsh C. 2004. Lessons from natural molecules. Nature New Biol 432:829–837. doi:10.1038/nature03194 15602548

[4] Newman DJ, Cragg GM. 2020. Natural products as sources of new drugs over the nearly four decades from 01/1981 to 09/2019. J Nat Prod 83:770–803. doi:10.1021/acs.jnatprod.9b01285 32162523

[5] Romsdahl J, Wang CCC. 2019. Recent advances in the genome mining of Aspergillus secondary metabolites (covering 2012-2018). Medchemcomm 10:840–866. doi:10.1039/c9md00054b 31303983 PMC6590338

[6] Zhgun AA. 2023. Fungal BGCs for production of secondary metabolites: main types, central roles in strain improvement, and regulation according to the piano principle. Int J Mol Sci 24:11184. doi:10.3390/ijms241311184 37446362 PMC10342363

[7] Han J, Jiang L, Zhang L, Quinn RJ, Liu X, Feng Y. 2021. Peculiarities of meroterpenoids and their bioproduction. Appl Microbiol Biotechnol 105:3987–4003. doi:10.1007/s00253-021-11312-z 33937926

[8] Keller NP. 2015. Translating biosynthetic gene clusters into fungal armor and weaponry. Nat Chem Biol 11:671–677. doi:10.1038/nchembio.1897 26284674 PMC4682562

[9] Bergmann S, Schümann J, Scherlach K, Lange C, Brakhage AA, Hertweck C. 2007. Genomics-driven discovery of PKS-NRPS hybrid metabolites from Aspergillus nidulans. Nat Chem Biol 3:213–217. doi:10.1038/nchembio869 17369821

[10] Baccile JA, Spraker JE, Le HH, Brandenburger E, Gomez C, Bok JW, Macheleidt J, Brakhage AA, Hoffmeister D, Keller NP, Schroeder FC. 2016. Plant-like biosynthesis of isoquinoline alkaloids in Aspergillus fumigatus. Nat Chem Biol 12:419–424. doi:10.1038/nchembio.2061 27065235 PMC5049701

[11] Chooi Y-H, Fang J, Liu H, Filler SG, Wang P, Tang Y. 2013. Genome mining of a prenylated and immunosuppressive polyketide from pathogenic fungi. Org Lett 15:780–783. doi:10.1021/ol303435y 23368997 PMC3576815

[12] Wang W, Yu Y, Keller NP, Wang P. 2021. Presence, mode of action, and application of pathway specific transcription factors in Aspergillus biosynthetic gene clusters. Int J Mol Sci 22:8709. doi:10.3390/ijms22168709 34445420 PMC8395729

[13] Lind AL, Lim FY, Soukup AA, Keller NP, Rokas A. 2018. An LaeA- and BrlA-dependent cellular network governs tissue-specific secondary metabolism in the human pathogen Aspergillus fumigatus. mSphere 3:e00050-18. doi:10.1128/mSphere.00050-18 29564395 PMC5853485

[14] Tudzynski B. 2014. Nitrogen regulation of fungal secondary metabolism in fungi. Front Microbiol 5:656. doi:10.3389/fmicb.2014.00656 25506342 PMC4246892

[15] Wiemann P, Lechner BE, Baccile JA, Velk TA, Yin WB, Bok JW, Pakala S, Losada L, Nierman WC, Schroeder FC, Haas H, Keller NP. 2014. Perturbations in small molecule synthesis uncovers an iron-responsive secondary metabolite network in Aspergillus fumigatus. Front Microbiol 5:530. doi:10.3389/fmicb.2014.00530 25386169 PMC4208449

[16] Carriel CC, Pyne S, Halberg-Spencer SA, Park SC, Seo H, Schmidt A, Calise DG, Ané J-M, Keller NP, Roy S. 2023. A network-based model of Aspergillus fumigatus elucidates regulators of development and defensive natural products of an opportunistic pathogen. bioRxiv. doi:10.1101/2023.05.11.538573 PMC12781895 41505094

[17] Dolan SK, O’Keeffe G, Jones GW, Doyle S. 2015. Resistance is not futile: gliotoxin biosynthesis, functionality and utility. Trends Microbiol 23:419–428. doi:10.1016/j.tim.2015.02.005 25766143

[18] de Castro PA, Colabardini AC, Moraes M, Horta MAC, Knowles SL, Raja HA, Oberlies NH, Koyama Y, Ogawa M, Gomi K, Steenwyk JL, Rokas A, Gonçales RA, Duarte-Oliveira C, Carvalho A, Ries LNA, Goldman GH. 2022. Regulation of gliotoxin biosynthesis and protection in Aspergillus species. PLoS Genet 18:e1009965. doi:10.1371/journal.pgen.1009965 35041649 PMC8797188

[19] Fabri J, Rocha MC, Fernandes CM, Persinoti GF, Ries LNA, da Cunha AF, Goldman GH, Del Poeta M, Malavazi I. 2021. The heat shock transcription factor HsfA is essential for thermotolerance and regulates cell wall integrity in Aspergillus fumigatus. Front Microbiol 12:656548. doi:10.3389/fmicb.2021.656548 33897671 PMC8062887

[20] Seo HW, Wassano NS, Amir Rawa MS, Nickles GR, Damasio A, Keller NP. 2024. A timeline of biosynthetic gene cluster discovery in Aspergillus fumigatus: from characterization to future perspectives. J Fungi (Basel) 10:266. doi:10.3390/jof10040266 38667937 PMC11051388

[21] Yin WB, Baccile JA, Bok JW, Chen Y, Keller NP, Schroeder FC. 2013. A nonribosomal peptide synthetase-derived iron(III) complex from the pathogenic fungus Aspergillus fumigatus. J Am Chem Soc 135:2064–2067. doi:10.1021/ja311145n 23360537 PMC3590312

[22] Raffa N, Won TH, Sukowaty A, Candor K, Cui C, Halder S, Dai M, Landero-Figueroa JA, Schroeder FC, Keller NP. 2021. Dual-purpose isocyanides produced by Aspergillus fumigatus contribute to cellular copper sufficiency and exhibit antimicrobial activity. Proc Natl Acad Sci U S A 118:e2015224118. doi:10.1073/pnas.2015224118 33593906 PMC7923669

[23] Lee MK, Kwon NJ, Choi JM, Lee IS, Jung S, Yu JH. 2014. NsdD is a key repressor of asexual development in Aspergillus nidulans. Genetics 197:159–173. doi:10.1534/genetics.114.161430 24532783 PMC4012476

[24] Lee MK, Kwon NJ, Lee IS, Jung S, Kim SC, Yu JH. 2016. Negative regulation and developmental competence in Aspergillus. Sci Rep 6:28874. doi:10.1038/srep28874 27364479 PMC4929475

[25] Szewczyk E, Krappmann S. 2010. Conserved regulators of mating are essential for Aspergillus fumigatus cleistothecium formation. Eukaryot Cell 9:774–783. doi:10.1128/EC.00375-09 20348388 PMC2863953

[26] Lim FY, Won TH, Raffa N, Baccile JA, Wisecaver J, Rokas A, Schroeder FC, Keller NP. 2018. Fungal isocyanide synthases and xanthocillin biosynthesis in Aspergillus fumigatus. mBio 9:e00785-18. doi:10.1128/mBio.00785-18 29844112 PMC5974471

[27] Medema MH, Fischbach MA. 2015. Computational approaches to natural product discovery. Nat Chem Biol 11:639–648. doi:10.1038/nchembio.1884 26284671 PMC5024737

[28] Chavali AK, Rhee SY. 2018. Bioinformatics tools for the identification of gene clusters that biosynthesize specialized metabolites. Brief Bioinformatics 19:1022–1034. doi:10.1093/bib/bbx020 28398567 PMC6171489

[29] Blin K, Kim HU, Medema MH, Weber T. 2019. Recent development of antiSMASH and other computational approaches to mine secondary metabolite biosynthetic gene clusters. Brief Bioinformatics 20:1103–1113. doi:10.1093/bib/bbx146 29112695 PMC6781578

[30] van Dijk JWA, Wang CCC. 2016. Heterologous expression of fungal secondary metabolite pathways in the Aspergillus nidulans host system. Methods Enzymol 575:127–142. doi:10.1016/bs.mie.2016.02.021 27417927

[31] Steenwyk JL, Mead ME, Knowles SL, Raja HA, Roberts CD, Bader O, Houbraken J, Goldman GH, Oberlies NH, Rokas A. 2020. Variation among biosynthetic gene clusters, secondary metabolite profiles, and cards of virulence across Aspergillus species. Genetics 216:481–497. doi:10.1534/genetics.120.303549 32817009 PMC7536862

[32] Bok JW, Keller NP. 2004. LaeA, a regulator of secondary metabolism in Aspergillus spp. Eukaryot Cell 3:527–535. doi:10.1128/EC.3.2.527-535.2004 15075281 PMC387652

[33] Gardiner DM, Howlett BJ. 2005. Bioinformatic and expression analysis of the putative gliotoxin biosynthetic gene cluster of Aspergillus fumigatus. FEMS Microbiol Lett 248:241–248. doi:10.1016/j.femsle.2005.05.046 15979823

[34] Schrettl M, Carberry S, Kavanagh K, Haas H, Jones GW, O’Brien J, Nolan A, Stephens J, Fenelon O, Doyle S. 2010. Self-protection against gliotoxin--A component of the gliotoxin biosynthetic cluster, GliT, completely protects Aspergillus fumigatus against exogenous gliotoxin. PLoS Pathog 6:e1000952. doi:10.1371/journal.ppat.1000952 20548963 PMC2883607

[35] Bok JW, Chung D, Balajee SA, Marr KA, Andes D, Nielsen KF, Frisvad JC, Kirby KA, Keller NP. 2006. GliZ, a transcriptional regulator of gliotoxin biosynthesis, contributes to Aspergillus fumigatus virulence. Infect Immun 74:6761–6768. doi:10.1128/IAI.00780-06 17030582 PMC1698057

[36] Scharf DH, Remme N, Heinekamp T, Hortschansky P, Brakhage AA, Hertweck C. 2010. Transannular disulfide formation in gliotoxin biosynthesis and its role in self-resistance of the human pathogen Aspergillus fumigatus. J Am Chem Soc 132:10136–10141. doi:10.1021/ja103262m 20593880

[37] Dolan SK, Owens RA, O’Keeffe G, Hammel S, Fitzpatrick DA, Jones GW, Doyle S. 2014. Regulation of nonribosomal peptide synthesis: bis-thiomethylation attenuates gliotoxin biosynthesis in Aspergillus fumigatus. Chem Biol 21:999–1012. doi:10.1016/j.chembiol.2014.07.006 25126990

[38] Alves de Castro P, Figueiredo Pinzan C, Dos Reis TF, Valero C, Van Rhijn N, Menegatti C, de Freitas Migliorini IL, Bromley M, Fleming AB, Traynor AM, Sarikaya-Bayram Ö, Bayram Ö, Malavazi I, Ebel F, Barbosa JCJ, Fill T, Pupo MT, Goldman GH. 2024. Aspergillus fumigatus mitogen-activated protein kinase MpkA is involved in gliotoxin production and self-protection. Nat Commun 15:33. doi:10.1038/s41467-023-44329-1 38167253 PMC10762094

[39] Ries LNA, Pardeshi L, Dong Z, Tan K, Steenwyk JL, Colabardini AC, Ferreira Filho JA, de Castro PA, Silva LP, Preite NW, Almeida F, de Assis LJ, Dos Santos RAC, Bowyer P, Bromley M, Owens RA, Doyle S, Demasi M, Hernández DCR, Netto LES, Pupo MT, Rokas A, Loures FV, Wong KH, Goldman GH. 2020. The Aspergillus fumigatus transcription factor RglT is important for gliotoxin biosynthesis and self-protection, and virulence. PLoS Pathog 16:e1008645. doi:10.1371/journal.ppat.1008645 32667960 PMC7384679

[40] Shin KS, Park HS, Kim YH, Yu JH. 2013. Comparative proteomic analyses reveal that FlbA down-regulates gliT expression and SOD activity in Aspergillus fumigatus. J Proteomics 87:40–52. doi:10.1016/j.jprot.2013.05.009 23689084

[41] Dolan SK, Bock T, Hering V, Owens RA, Jones GW, Blankenfeldt W, Doyle S. 2017. Structural, mechanistic and functional insight into gliotoxin bis-thiomethylation in Aspergillus fumigatus. Open Biol 7:160292. doi:10.1098/rsob.160292 28179499 PMC5356443

[42] Ma D, Li R. 2013. Current understanding of HOG-MAPK pathway in Aspergillus fumigatus. Mycopathologia 175:13–23. doi:10.1007/s11046-012-9600-5 23161019

[43] Grosse V, Krappmann S. 2008. The asexual pathogen aspergillus fumigatus expresses functional determinants of Aspergillus nidulans sexual development. Eukaryot Cell 7:1724–1732. doi:10.1128/EC.00157-08 18757566 PMC2568067

[44] Shimizu K, Keller NP. 2001. Genetic involvement of a cAMP-dependent protein kinase in a G protein signaling pathway regulating morphological and chemical transitions in Aspergillus nidulans. Genetics 157:591–600. doi:10.1093/genetics/157.2.591 11156981 PMC1461531

[45] Calvo AM, Bok J, Brooks W, Keller NP. 2004. veA is required for toxin and sclerotial production in Aspergillus parasiticus. Appl Environ Microbiol 70:4733–4739. doi:10.1128/AEM.70.8.4733-4739.2004 15294809 PMC492383

[46] Bok JW, Soukup AA, Chadwick E, Chiang Y-M, Wang CCC, Keller NP. 2013. VeA and MvlA repression of the cryptic orsellinic acid gene cluster in Aspergillus nidulans involves histone 3 acetylation. Mol Microbiol 89:963–974. doi:10.1111/mmi.12326 23841751 PMC3773851

[47] Soukup AA, Farnoodian M, Berthier E, Keller NP. 2012. NosA, a transcription factor important in Aspergillus fumigatus stress and developmental response, rescues the germination defect of a laeA deletion. Fungal Genet Biol 49:857–865. doi:10.1016/j.fgb.2012.09.005 23022264 PMC3483426

