# Supplementary material for: Gene regulatory network resource aids in predicting trans-acting regulators of biosynthetic gene clusters in Aspergillus fumigatus
Source: mBio. 2025 Feb 18;16(3):e03874-24. doi: 10.1128/mbio.03874-24 (PMC11898546; doi:10.1128/mbio.03874-24)
Supplement: Supplemental figures — Fig. S1 to S8. [file mbio.03874-24-s0001.pdf]

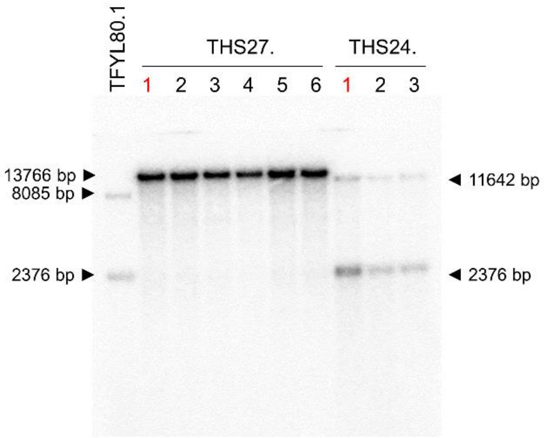

Figure S1. Southern confirmation of Knock-down strain of *hsfA* (KD*hsfA*, THS27) and overexpression strain of *hsfA* (Oe*hsfA*, THS24) in *A. fumigatus*.

Genomic DNA was digested by *Bst*XI producing two bands with 8085 bp and 2376 bp from TFYL80.1, one band with 13766 bp from p*xylP::hsfA*(AFUA_5G01900 (KD*hsfA*, THS27), and two bands with 11642 and 2376 bp from *pyrG*::p*gpdA*::*hsfA* (OE*hsfA,* THS24). THS27.1 and THS24.1 were chosen for the subsequent experiments.

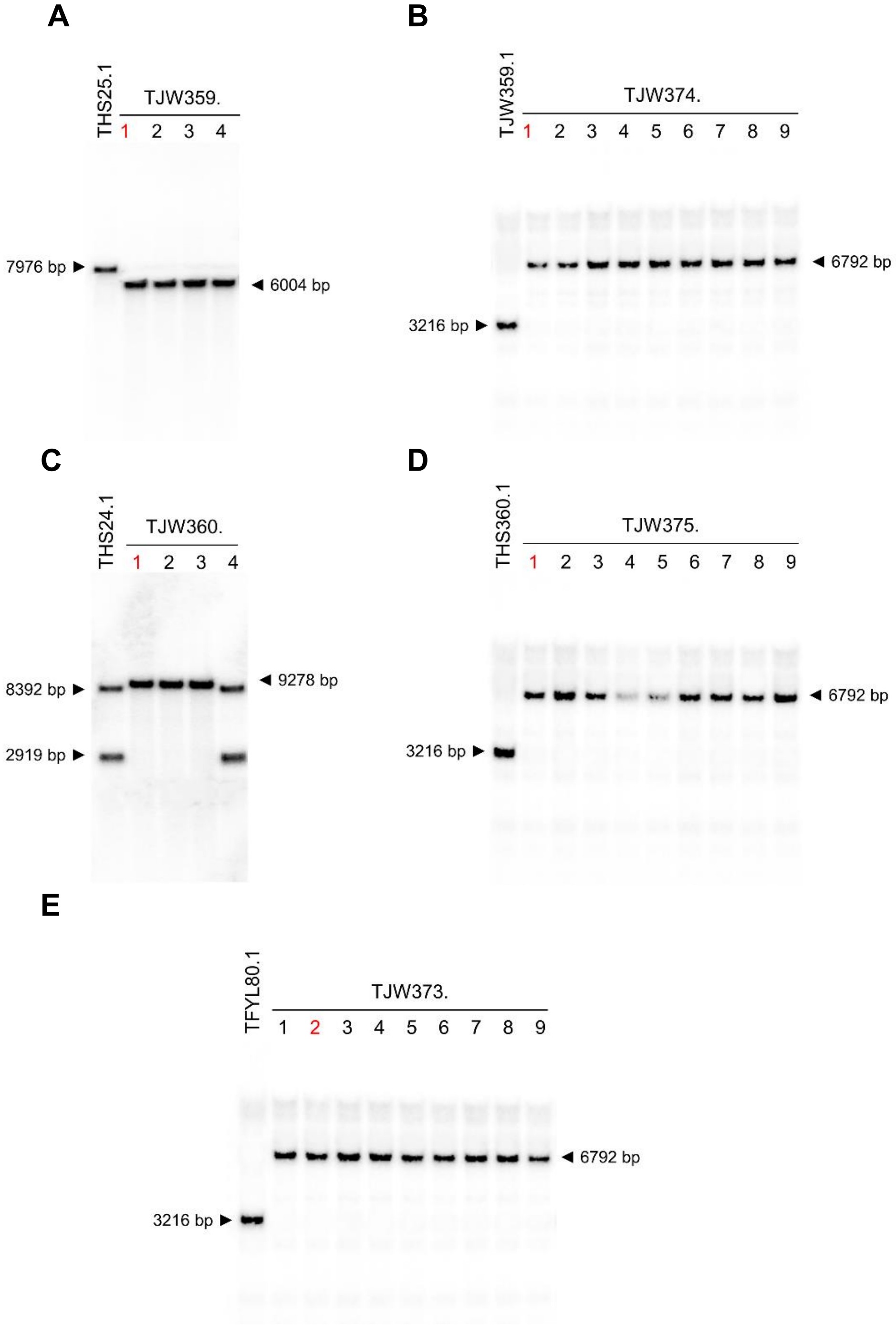

Figure S2. Southern confirmation of OE*rogA,pyrG-* (TJW359), OE*hsfA,pyrG-* (TJW360), OE*rogA*OE*gliZ* (TJW374), OE*hsfA*OE*gliZ* (TJW375) and OE*gliZ* (TJW373).

A. Southern blot for confirmation of recycling *pyrG* from OE*rogA*. Genomic DNA was digested by *Nde*I producing one band with 7976 bp from OE*rogA* (THS25.1) and one band with 6004 bp from OE*rogA-pyrG* (TJW359). TJW359.1 was chosen for the subsequent experiments. **B. Sounthern blot for confirmation of OE*rogA*OE*gliZ*.** Genomic DNA was digested by *Sac*II producing one band with 3216 bp from TJW359.1 and one band with 6792 bp from OE*rogA*OE*gliZ* (TJW374). TJW374.1 was chosen for the subsequent experiments. **C. Southern blot for confirmation of recycling *pyrG* from OE*hsfA*.** Genomic DNA was digested by *Nco*I producing two band with 2919 bp and 8392 bp from OE*hsfA* (THS24.1) and one band with 9278 bp from OE*hsfA-pyrG* (TJW360). TJW360.1 was chosen for the subsequent experiments. **D. Sounthern blot for confirmation of OE*hsfA*OE*gliZ*.** Genomic DNA was digested by *Sac*II producing one band with 3216 bp from TJW360.1 and one band with 6792

bp from OE*rogA*OE*gliZ* (TJW375). TJW375.1 was chosen for the subsequent experiments. **E. Sounthern blot for confirmation of OE*gliZ*.** Genomic DNA was digested by *Sac*II producing one band with 3216 bp from TFYL80.1 and one band with 6792 bp from OE*gliZ* (TJW373). TJW373.2 was chosen for the subsequent experiments.

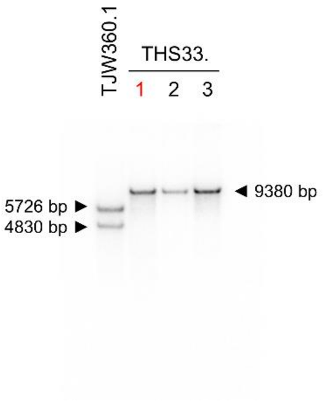

Figure S3. Southern confirmation of OE*hsfA*Δ*rogA* double mutant (THS33) in *A. fumigatus*.

Genomic DNA was digested by *Nde*I producing two bands with 4830 bp and 5726 bp from TJW360.1 (OE*hsfA*-*pyrG*), and one band with 9380 bp from p*xylP::hsfA*, Δ*rogA::pyrG* (OE*hsfA*Δ*rogA*, THS33). THS33.1 were chosen for the subsequent experiments.

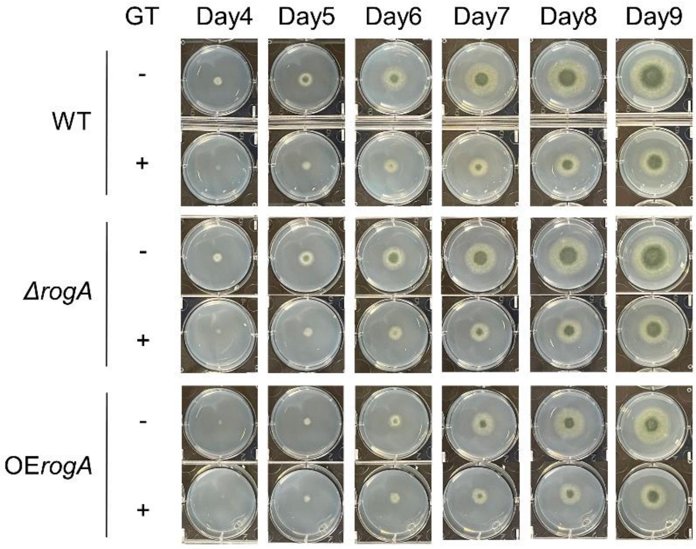

Figure S4. Gliotoxin resistance of wild-type, Δ*rogA* and OE*rogA.*

5X10^3 spores of wild-type, Δ*rogA* and OE*rogA* mutants of *A. fumigatus* were inoculated on glucose minimum media (GMM) with or without 50μg/ml gliotoxin (GT). The cells were incubated at 25°C for 9 days in the dark. The GT-treated cells started to grow 4 days after the inoculation. All these experiments were performed in triplicate.

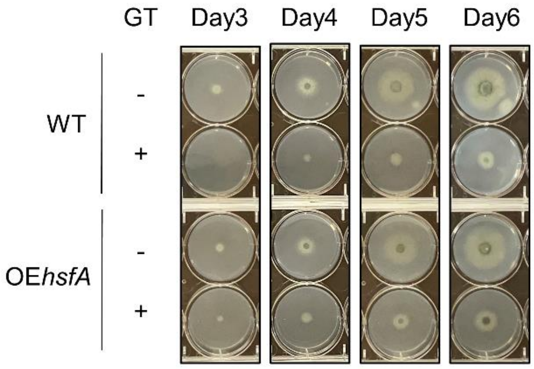

Fig. S5. Gliotoxin resistance of wild-type and OE *hsfA* mutants.

5X10^{3} spores of wild-type and OE*hsfA* of *A. fumigatus* were inoculated on glucose minimum media (GMM) with or without 50µg/ml gliotoxin (GT). The cells were incubated at 25°C for 6 days in the dark. The GT-treated OE*hsfA* started growing at 3 days after the inoculation, while GT-treated WT at 4 days after the inoculation. All these experiments were performed in triplicate.

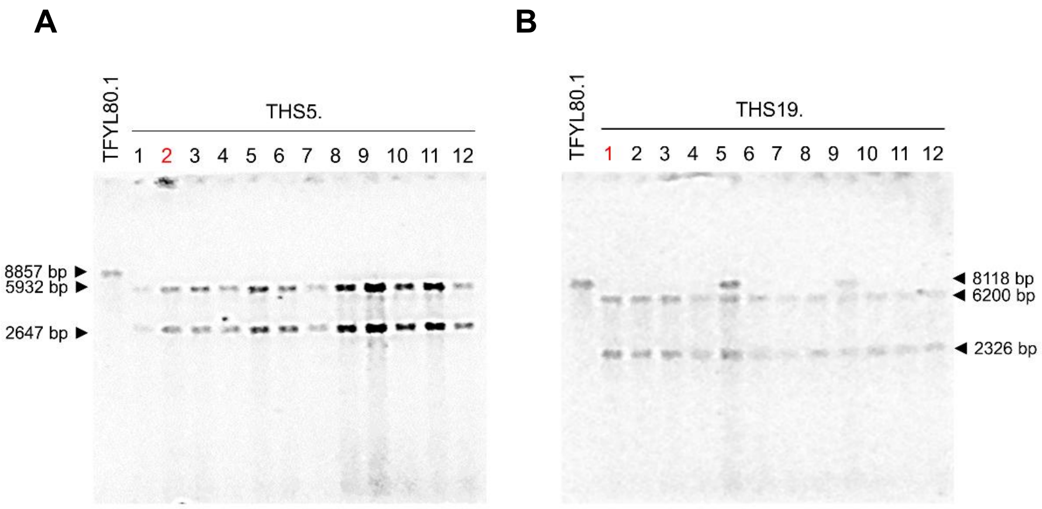

Figure S6. Southern confirmation of Δ*skn7* (THS5) and OE*skn7* (THS19) in *A. fumigatus*.

A. Genomic DNA was digested by *Nhe*I producing one band with 8857 bp from TFYL80.1, and two bands with 5932 and 2647 bp from Δ*skn7::pyrG* (Δ*skn7* (AFUA_6G12522), THS5). THS33.2 were chosen for the subsequent experiments. **B.** Genomic DNA was digested by *Sal*I producing one band with 8118 bp from TFYL80.1, and two bands with 6200 and 2326 bp from p*gpdA::skn7* (OE*skn7*, THS19). THS19.1 were chosen for the subsequent experiments.

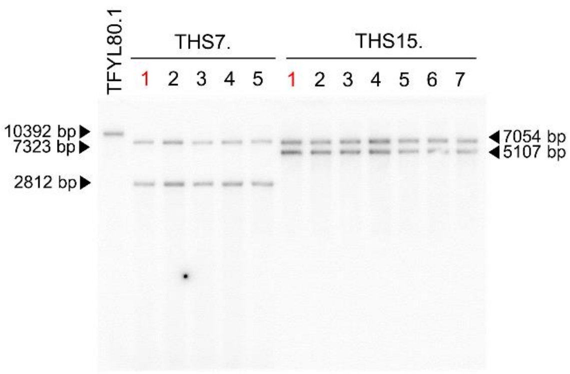

Figure S7. Southern confirmation of Δ*rofA* (THS7) and OE*rofA* (THS15) in *A. fumigatus*.

Genomic DNA was digested by *Kpn*I producing one band with 10392 bp from TFYL80.1, two bands with 7323 and 2812 bp from Δ*rofA::pyrG* (Δ*rofA* (AFUA_1G14945), THS5), and two bands with 7054, 2812 bp from p*gpdA::rofA* (OE*rofA*, THS15). THS7.1 and THS15.1 were chosen for the subsequent experiments.

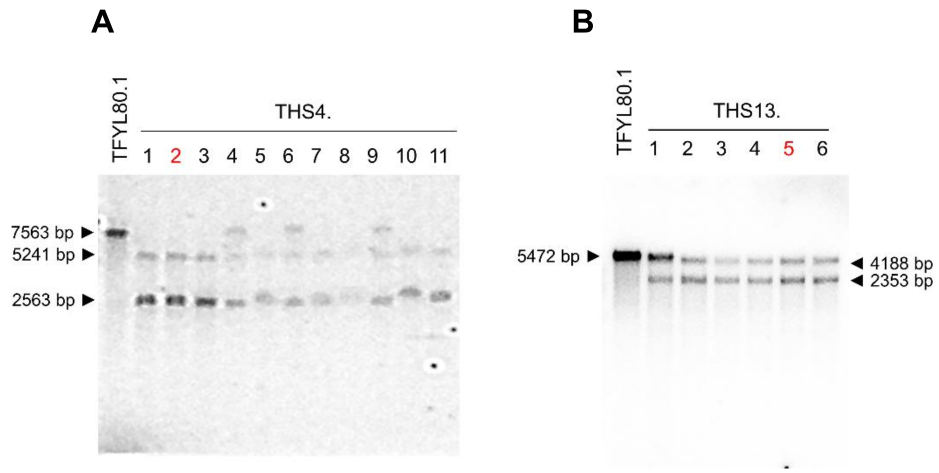

Figure S8. Southern confirmation of Δ*nsdD* (THS4) and OE*nsdD* (THS13) in *A. fumigatus*.

A. Genomic DNA was digested by *Sal*I producing one band with 7563 bp from TFYL80.1, and two bands with 5241 and 2563 bp from Δ*nsdD::pyrG* (Δ*nsdD* (AFUA_3G13870), THS4). THS5.2 were chosen for the subsequent experiments. **B.** Genomic DNA was digested by *Nco*I producing one band with 5472 bp from TFYL80.1, and two bands with 4188 and 2353 bp from p*gpdA::nsdD* (OE*nsdD*, THS13). THS13.5 were chosen for the subsequent experiments.
